# Les soins palliatifs en hématologie: quel avenir en Afrique?

**DOI:** 10.4314/pamj.v8i1.71055

**Published:** 2011-02-10

**Authors:** Illias Tazi, Hatim Nafil, Lahoucine Mahmal

**Affiliations:** 1 Service d’hématologie, CHU Mohamed VI, Université Cadi Ayyad, Marrakech, Maroc

**Keywords:** Hématologie, soins palliatifs, Afrique

## Abstract

Les hémopathies malignes sont des maladies graves qui touchent de nombreux individus à travers le monde. Elles marquent l’entrée dans un lourd et long processus de soins. Le continent africain n’en est pas épargné. La lutte contre ce fléau en Afrique est confortée à de nombreux défis, malgré l’absence ou l’insuffisance des ressources matérielles et financières, et du manque de personnel qualifié. Malgré les multiples insuffisances en structures de soins adaptées et de ressources limitées, l’idée de penser à développer les soins palliatifs arrive à temps. Cela ne demande qu’une bonne formation des soignants d’une part et une bonne volonté des praticiens d’une autre part. Comme les soins palliatifs visent la globalité des besoins de la personne en fin de vie et concernent également son entourage, il est primordial que les interventions des différents prestataires s’agencent correctement et résultent en une prise en charge globale et efficace.

## Lettre aux éditeurs

Les hémopathies malignes sont des maladies graves qui touchent de nombreux individus à travers le monde. Elles marquent l’entrée dans un lourd et long processus de soins. Le continent africain n’en est pas épargné. La lutte contre ce fléau en Afrique est confortée à de nombreux défis, malgré l’absence ou l’insuffisance des ressources matérielles et financières, et du manque de personnel qualifié. L’hématologie a connu ces dernières années d’importants progrès dans le traitement des hémopathies malignes surtout concernant la leucémie myéloïde chronique, le myélome multiple et les lymphomes non Hodgkiniens avec une augmentation de la survie globale et la survie sans événement. De même chez l’enfant, le traitement de la maladie d’Hodgkin et des leucémies aigues lymphoblastiques a connu une augmentation importante du taux de guérison. Toutefois, le patients présentant une hémopathie maligne, vont atteindre à un moment donné une phase terminale de la maladie qui échappe à toute ressource thérapeutique [1]. Il nous revient donc d’améliorer la qualité de la prise en charge des malades et en particulier ceux qui n’ont pas les moyens pour se faire soigner, ceux qui sont isolés et ceux que l’on ne peut plus guérir. L’organisation des soins palliatifs doit être aujourd’hui une des priorités de l’Afrique tant que les moyens de diagnostic, de dépistage et de traitement ne sont pas encore suffisamment développés.

Dans la majorité des pays africains, les unités de soins palliatifs, y compris la gestion de la douleur et des symptômes, sont très limitées ou n’existent pas. Les soins palliatifs est une philosophie de soins qui associe toute gamme de thérapies dont l’objectif est d’assurer la meilleure qualité de vie possible aux patients souffrants de maladies mortelles et incurables ainsi qu’à leur famille. Cette philosophie est basée sur le principe que chacun a le droit d’être soigné et de mourir dans la dignité et que le soulagement de la douleur –physique, affective, spirituelle et socialeest un droit humain inaliénable et essentiel à ce processus. Les personnels de santé et les décideurs ignorent souvent qu’il existe des méthodes peu couteuses et efficaces pour soulager la douleur [2]. Au contraire, les ressources consacrées aux soins de santé sont trop souvent utilisées pour des traitements curatifs couteux. Les soins palliatifs, y compris le soulagement des symptômes, devraient être instaurés le plus tôt possible dans l’évolution d’une hémopathie maligne. Les soins palliatifs soutiennent la vie et considèrent que la mort est un processus normal. Ils ne cherchent ni à accélérer, ni à repousser la mort. Les programmes sur les soins palliatifs en hématologie, conjointement avec des programmes plus larges sur la cancer, doivent prendre en considération la promotion des informations sur les soins palliatifs adaptées aux décideurs et personnels de santé , étudier la politique médicale/pharmaceutique qui peut limiter inutilement l’accès à des médicaments appropriés, organiser des systèmes de soutien pour aider à mobiliser les ressources, former les personnels de santé aux principes de soins palliatifs à domicile.

Dans la majorité des pays d’Afrique, les programmes de prévention et de lutte contre le cancer font défaut ou présentent des insuffisances. Certes les soignants seront confrontés à de multiples problèmes, mais leur détermination doit être plus forte pour en faire face. Cela demande beaucoup de communication pour aider ces malades en grande souffrance. Les soins palliatifs sont l’affaire de tous les soignants et médecins hématologues qui ont à être attentifs aux malades et pas seulement à la maladie. Elle concerne autant les patients (et/ou leur famille) que les équipes soignantes. Un autre aspect de la question concerne la formation des différents acteurs de soins palliatifs qui est très faible d’où la nécessité de l’intégrer dans les programmes de formation des médecins généralistes ou spécialistes hématologues. Il faut donc offrir une formation aux soins palliatifs au personnel de santé des hôpitaux et de la communauté, aux enseignants et aux responsables communautaires; ceux-ci pourront à leur tour transmettre leur savoir aux agents de santé communautaire, aux bénévoles et aux familles.

Répondre à la demande des patients, à toutes les phases de la maladie hématologique suppose: l'identification des besoins de ces patients, une organisation pluridisciplinaire, transdisciplinaire et une articulation établissements de soins de médecine de ville, mais aussi, une responsabilisation des patients. Dans les pays développés, approximativement 50 % des patients atteints de cancer ne sont pas soulagés de leurs souffrances en raison de traitements inadaptés ou inexistants, et il est vraisemblable que ce nombre est beaucoup plus élevé dans les pays en développement. Mais les recherches ont montré que 70 à 90 % de la douleur due au cancer peut être soulagée même avec des ressources limitées, ce qui améliore largement la qualité de vie du patient [3]. Il revient donc à chaque pays de parfaire l’analyse de sa propre situation afin de produire et de développer les ressources humaines adéquates. Des efforts doivent être entrepris pour mieux développer cet aspect de l’hématologie. Ce qui implique bien sûr une forte volonté politique des décideurs pour arriver à cette fin.

Le rôle des organisations non gouvernementales (ONG) ne peut être que bénéfique en aidant les malades nécessiteux par un soutien financier, matériel et psychique. Malgré les multiples insuffisances en structures de soins adaptées et de ressources limitées, l’idée de penser à développer les soins palliatifs arrive à temps. Cela ne demande qu’une bonne formation des soignants d’une part et une bonne volonté des praticiens d’une autre part. Comme les soins palliatifs visent la globalité des besoins de la personne en fin de vie et concernent également son entourage, il est primordial que les interventions des différents prestataires s’agencent correctement et résultent en une prise en charge globale et efficace.

## Conflits d’intérêt

Les auteurs ne déclarent aucun conflit d’intérêts
